# Phenotyping Plant Responses to Biotic Stress by Chlorophyll Fluorescence Imaging

**DOI:** 10.3389/fpls.2019.01135

**Published:** 2019-09-18

**Authors:** María Luisa Pérez-Bueno, Mónica Pineda, Matilde Barón

**Affiliations:** Department of Biochemistry and Molecular and Cell Biology of Plants, Estación Experimental del Zaidín, Consejo Superior de Investigaciones Científicas, Granada, Spain

**Keywords:** photosynthesis, quenching, plant pathogen, laser-induced fluorescence, sun-induced fluorescence, hyperspectral reflectance, vegetation indices

## Abstract

Photosynthesis is a pivotal process in plant physiology, and its regulation plays an important role in plant defense against biotic stress. Interactions with pathogens and pests often cause alterations in the metabolism of sugars and sink/source relationships. These changes can be part of the plant defense mechanisms to limit nutrient availability to the pathogens. In other cases, these alterations can be the result of pests manipulating the plant metabolism for their own benefit. The effects of biotic stress on plant physiology are typically heterogeneous, both spatially and temporarily. Chlorophyll fluorescence imaging is a powerful tool to mine the activity of photosynthesis at cellular, leaf, and whole-plant scale, allowing the phenotyping of plants. This review will recapitulate the responses of the photosynthetic machinery to biotic stress factors, from pathogens (viruses, bacteria, and fungi) to pests (herbivory) analyzed by chlorophyll fluorescence imaging both at the lab and field scale. Moreover, chlorophyll fluorescence imagers and alternative techniques to indirectly evaluate photosynthetic traits used at field scale are also revised.

## Introduction

Changes in red chlorophyll *a* fluorescence (Chl-F) emission after illumination of dark-adapted plants with photosynthetically active radiance (PAR) were first reported by Kautsky and Hirsch (1931). This Chl-F showed a high correlation with photosynthetic rates. Ever since then, this technique has been heavily exploited to monitor photosynthetic performance and stress in plants. Indeed, no investigation regarding the activity of photosynthesis seems complete without some Chl-F data (Baker, 2008; Murchie and Lawson, 2013). Moreover, the sensitivity of Chl-F to even minor alterations in plant metabolism makes this technique suitable to provide insight into plant-stress factor interactions. Finally, the development of instruments capable of imaging Chl-F has provided a powerful tool to resolve spatial heterogeneity of plant and leaf photosynthetic performance under stress conditions. Chlorophyll fluorescence imaging (Chl-FI) makes available a wealth of information on the timing and location of pathogen development as well as to understand the regulation of photosynthesis from leaf to crop scale (Rolfe and Scholes, 2010). Moreover, Chl-FI is often included in phenomics together with metabolomics, genomics, transcriptomics, and proteomics. Indeed, Mir et al. (2019) have recently reviewed the high-throughput phenotyping platforms equipped with Chl-FI devices.

The study of Chl-F kinetics provides information on the efficiency of photosystem II (PSII) following the model proposed by Butler (1978). Basically, the model establishes that photochemistry (the so-called photochemical quenching) competes with the processes of energy dissipation as Chl-F and heat (the so-called non-photochemical quenching) for excitation energy in the antenna pigments of PSII. Thus, to analyze the photosynthetic performance of a sample from Chl-FI measurements, it is necessary to differentiate between the photochemical and non-photochemical components of quenching. The usual approach is to transiently reduce to zero the photochemistry component using saturating light flashes, so that the Chl-FI yield in the presence of the non-photochemical quenching alone can be estimated (Maxwell and Johnson, 2000).

The most commonly used Chl-F parameters related with photosynthetic activity are summarized in **Table 1**. In general terms, plants respond to stress conditions activating acclimation mechanisms to adjust the machinery to the new environment with the aim of maintaining the photosynthetic activity. It might include the increase in the capacity for energy dissipation, detected by increases in non-photochemical quenching (measured as NPQ and qN) with no alterations in maximum quantum efficiency of PSII (F_V_/F_M_). When the stress overcomes the capacity of acclimation, permanent photoinhibition occurs and can be detected by decreases in F_V_/F_M_. When stress is strong or prolonged enough, the effective quantum yield of PSII (Φ_PSII_) and the photochemical quenching (qP) decrease, meaning an inhibition of the electron transport chain. This inhibition of the light-dependent reactions can be accompanied by an increase in NPQ and qN. However, severe stress conditions might cause a severe loss of functionality of the PSII, and these three parameters would decrease (Horton et al., 2008; Murchie and Lawson, 2013). In the following sections, alterations in Chl-F parameters for individual host-pathogen systems will be described in more detail.

**Table 1:** Chl-F parameters of common use in biotic stress detection. For further details, see Roháček and Barták (1999), Maxwell and Johnson (2000), and Murchie and Lawson (2013).

Chl-F parameters	Known as	Formula
Maximum quantum yield of PSII	F_V_/F_M_	(F_M_ − F_0_)/F_M_
Effective quantum yield of PSII	Φ_PSII_	(F’_M_ − F_t_)/F’_M_
Photochemical quenching	qP	(F’_M_ − F_t_)/(F’_M_ − F’_0_)
Non-photochemical quenching	NPQ	(F_M_ − F’_M_)/F’_M_
	qN	1 − (F’_M_ − F’_0_)/(F_M_ − F_0_)

This review addresses: (i) the different responses of the photosynthetic machinery to biotic stress factors—pathogens (viruses, bacteria, and fungi) and pests (herbivory and parasites)—analyzed by Chl-FI at lab scale and (ii) the different techniques based on Chl-F and alternatives that have been developed to investigate the photosynthetic performance in the field, with application to the diagnosis of biotic stress. Finally, prospects of Chl-F-based technologies applied to remote sensing and crop protection are discussed.

## Biotic Stress Detection at Lab Scale

### Viruses

The study of the timing and location of viral diseases in host plants was one of the first applications of Chl-FI (Balachandran et al., 1994). Hence, a number of studies have also used Chl-FI to evaluate the effect of viruses on primary metabolism, linking those findings to other alterations in plant physiology. Photoinhibitory damage of symptomatic tissues but also in asymptomatic areas of the infected plants during pathogenesis has been widely demonstrated. Moreover, Chl-FI can be used for diagnostic purposes before the appearance of visible symptoms (Barón et al., 2016). It was the case of *Tobacco mosaic virus* (TMV)–infected tobacco plants (Chaerle et al., 2007). Before symptoms appeared, affected areas of infected leaves showed low Chl-F values and little quenching capacity upon exposure to actinic light. These regions subsequently showed chlorotic–mosaic symptoms induced by TMV (Balachandran et al., 1994). Zucchini cotyledons inoculated with *Cucumber mosaic virus* (CMV) also showed different regions with different intensities of Chl-F emission before symptoms appearance (Técsi et al., 1994). Authors demonstrated that regions with high ability of Chl-F quenching correlated with leaf areas where starch would accumulate 24 h later, thus concluding that those areas possessed high qP capacity. On the contrary, *Abutilon striatum* leaves infected with *Abutilon mosaic virus* showed impaired NPQ in infected tissues that accompanied symptom expansion rather than underlying alterations in plant carbohydrate status (Osmond et al., 1998; Lohaus et al., 2000). On the other hand, tobacco plants resistant to TMV displayed a presymtompatic increment of Chl-F which evolved to a lower intensity patch surrounded by a high Chl-F intensity halo. This leaf area corresponded to the region where a visible hypersensitive response (HR) developed later on (Chaerle et al., 2004). Moreover, the damage caused by the reactive oxygen species (ROS) in soybean leaves infected with *Soybean mosaic virus* spatially correlated with decreases in Φ_PSII_, showing a negative trend of their respective relationship, as well (Aldea et al., 2006a). An spatial correlation between the Chl-FI (either increased NPQ or Chl-F at high excitation light) pattern and viral distribution was also found in asymptomatic *Nicotiana benthamiana* leaves infected with the Italian and the Spanish strains of the *Pepper mild mottle virus* (PMMoV) (Chaerle et al., 2006; Pérez-Bueno et al., 2006). This effect took place progressively in each downstream leaf invaded by PMMoV (Pineda et al., 2011). Therefore, Chl-FI proved to be an outstanding method for real-time tracking of viral movement in the host plant. In contrast, symptomatic leaves of pepper plants harboring the L3 resistant gene and inoculated with PMMoV showed decreased values of NPQ. The inoculation of the same pepper plants with the *Obuda pepper virus* led to an increase in F_V_/F_M_, Φ_PSII_, and NPQ in the areas adjacent to the infected ones (Rys et al., 2014). NPQ was also revealed as the most useful parameter to follow the infection caused by *Potato virus Y* in wild type and transgenic tobacco plants overproducing endogenous cytokinins; nevertheless, the viral-induced Chl-FI alterations were no presymptomatic in this pathosystem (Spoustova et al., 2013). Also coinciding with symptoms appearance, photosynthetic alterations in pea leaves infected by *Pea enation mosaic virus* consisted in a decrease of Φ_PSII_ together with an increase of NPQ (Kyseláková et al., 2011).

Chl-FI has also been used to assess the effect of mutations in the photosynthesis of virus-infected plants. Golden2-like (GLKs) transcription factors, with an unclear involvement in plant resistance to virus, play important roles in regulation of photosynthesis-associated nuclear genes in *Arabidopsis*, participating also in development of chloroplast. Double mutants of GLKs resulted more susceptible to CMV infection and showed decreased F_V_/F_M_ and Φ_PSII_ when compared either to the controls or to the single mutants. These results suggested that, in *Arabidopsis*, GLK1 and GLK2 might play redundant roles in virus resistance (Han et al., 2016). Similarly, the uncoupling protein (UCP), member of the plant mitochondrial energy dissipation pathway (which coordinates cellular energy metabolism), might play a role in the resistance to *Turnip crinkle virus* (TCV) infection in *Arabidopsis*. In fact, the decline in Φ_PSII_ values registered on TCV-infected wild-type plants were lower compared to the drop recorded when measuring the same parameter in *ucp1*- or *ucp2*-deficient TCV-infected plants (Pu et al., 2016).

**Figure 1:**
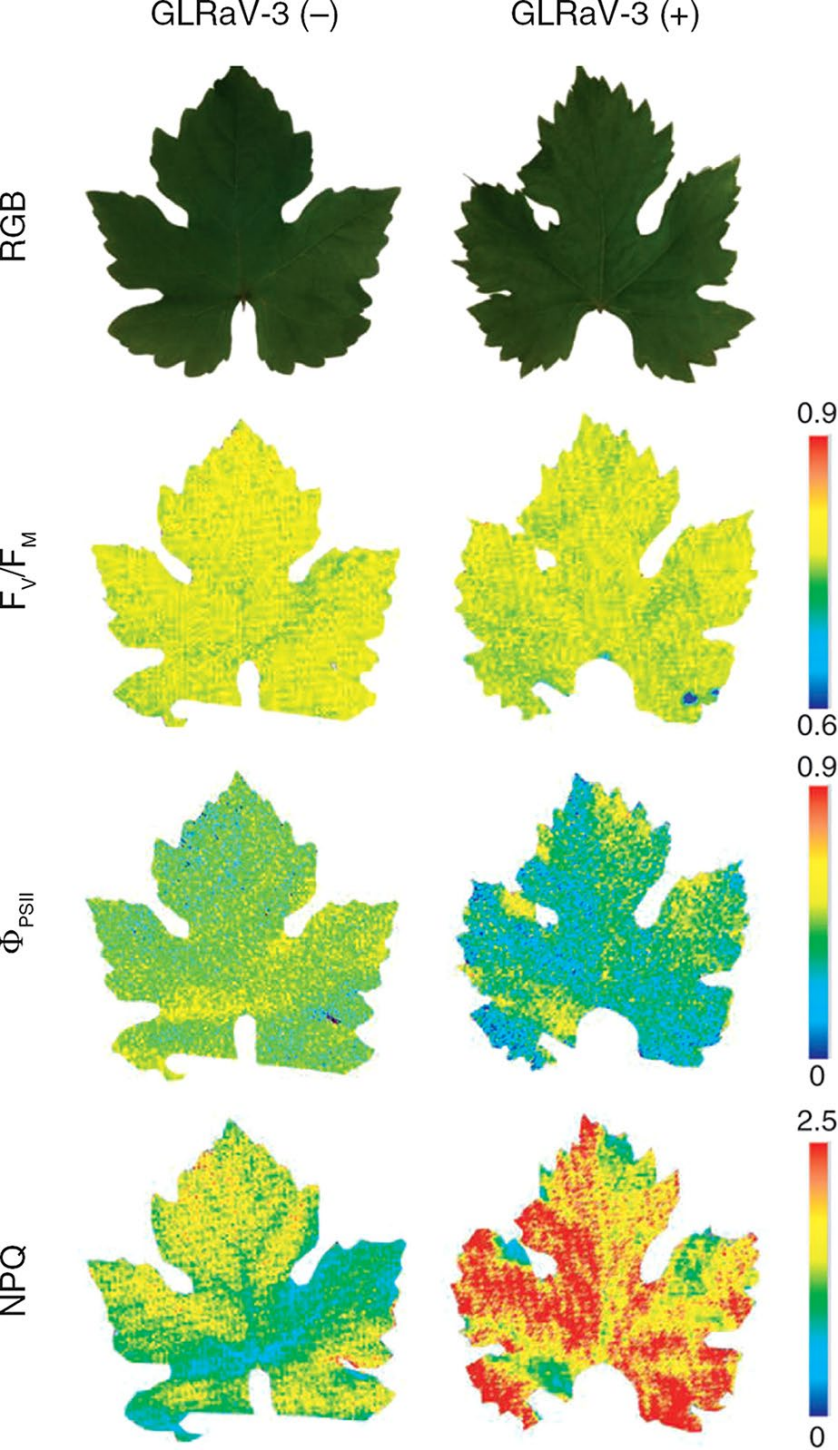
Impact of *Grapevine leafroll-associated virus 3* (GLRaV-3) infection on the photosynthesis of grapevine (*Vitis vinifera* white variety ‘Malvasía de Banyalbufar’) leaves. Representative images of PSII efficiency (F_V_/F_M_, Φ_PSII_) and non-photochemical quenching (NPQ) are shown. GLRaV-3 (−): control leaves; GLRaV-3(+): infected leaves; RGB: color reflectance pictures. Reproduced with permissions from Montero et al. (2016).

The combination of Chl-FI with other biochemical techniques can provide more insights about plant metabolism under viral infections. For example, metabolomics studies were carried out in combination with Chl-FI to estimate the alterations in the primary metabolism of grapevine upon infection with *Grapevine leafroll-associated virus 3* (Montero et al., 2016). The F_V_/F_M_, Φ_PSII_, and NPQ parameters showed photoinhibition of PSII in infected plants (**Figure 1**). Moreover, the increase in NPQ correlated with a decrease in the accumulation of some photorespiratory intermediates in infected plants. Several omics have also been used along with Chl-FI to evaluate the beneficial trade-offs from viral infections against drought (Aguilar et al., 2017).

Occasionally, standard Chl-F parameters do not always offer clear differences between healthy and infected tissues, or do so at late stages of the disease. In these cases, it is necessary to apply other mathematical tools to enhance such differences in the early stages of the infection. Combinatorial imaging analysis is an advance statistical approach rendering parameters with no physiological meaning, which in turn offers the highest contrast between treatments. As an example, combinatorial imaging revealed the infection caused by PMMoV in asymptomatic leaves of *N. benthamiana* plants earlier than standard Chl-FI parameters, even before than viral capsid can be detected by immunodetection in those leaves (Pineda et al., 2008).

### Bacteria

Although bacterial diseases cause important economic losses in agriculture worldwide, fewer works have applied Chl-FI to study the impact of bacterial infection, compared to other types of pathogens. A number of them are focused on the effect of virulent or avirulent pathovars of *Pseudomonas syringae*, causing systemic infection or HR, respectively. Soybean plants infected with an avirulent strain of *P. syringae* pv. *glycinea* showed lowered values of F_V_/F_M_ and Φ_PSII_, as well as an increase in NPQ, prior to the development of symptoms. However, little changes were observed in plants infected with a virulent strain (Zou et al., 2005). On the other hand, *Arabidopsis* plants displayed lower values of F_V_/F_M_, Φ_PSII_, and NPQ when infected with either a virulent or an avirulent *P. syringae* pv. *tomato* (Pto) strain (Bonfig et al., 2006). The same pathosystem was analyzed by combinatorial imaging to obtain images of Chl-FI parameters with no physiological meaning, but with high-resolving power to identify infected leaf areas earlier than Chl-FI standard parameters. The symptoms visualized by combinatorial imaging were stronger in the plants infected with the avirulent strain than in those inoculated with the virulent one. Moreover, the applied algorithms were also able to identify Chl-FI signatures characterizing early and late phases of the infection (Matouš et al., 2006; Berger et al., 2007). In bean plants, *P. syringae* pv. *phaseolicola* (Pph) causes a compatible infection, whereas Pto produces an HR. Using an inoculum concentration resembling those encountered in the field (10^4^ colony forming units [cfu]·ml^−1^), Pto-infected plants presented little Chl-FI changes respecting to the controls. However, NPQ maximized the differences between control and Pph-infected plants at 5 days post-inoculation (dpi), before symptoms appearance, in both infiltrated and non-infiltrated areas of the bean leaf. Moreover, the decrease in NPQ values in the non-infiltrated leaf areas inversely correlated to the cfu isolated from those leaf areas (Rodríguez-Moreno et al., 2008). However, when infiltrating leaves with a higher inoculum dose (10^7^ cfu·ml^−1^), alterations caused by Pto and Pph infections on bean photosynthesis could be detected at earlier time points: at 3 and 6 hours post-infection (hpi), respectively. Decreases in F_V_/F_M_ and Φ_PSII_ and increases in NPQ were measured at those time points. At later stages of the compatible infection, NPQ started to diminish in the inoculated areas, whereas the development of chlorosis in non-inoculated zones was preceded by increases in NPQ values. It was hypothesized that as the leaf tissue is progressively colonized by the pathogen, an increment of NPQ occurs, followed by a decline in the activity of the thylakoid as soon as the total viable count reaches a certain concentration (Pérez-Bueno et al., 2015).

**Figure 2:**
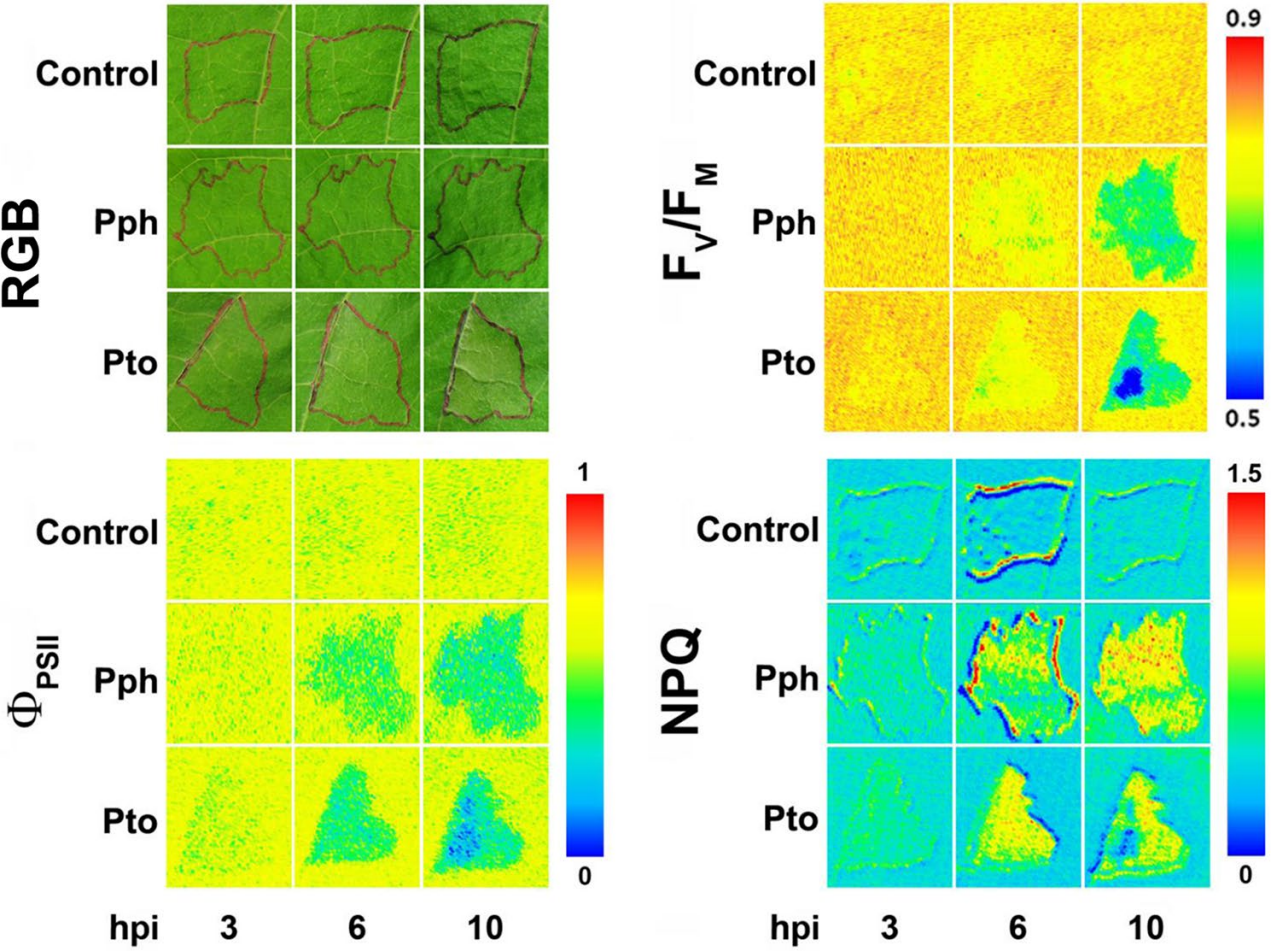
Symptoms evolution in bean leaves infiltrated either with *Pseudomonas syringae* pv. *phaseolicola* (Pph) or pv. *tomato* (Pto) at high dose (10^7^ cfu·ml^−1^), compared with control leaves (RGB panel). Infiltrated areas are marked in black. Representative measurements of short-term effect of bacterial infection on photosynthesis, analyzed in terms of PSII efficiency (F_V_/F_M_ and Φ_PSII_ panels) and non-photochemical quenching (NPQ panel). hpi, hours post-infection. Modified from Pérez-Bueno et al. (2015) with permissions.

The infection caused by the necrotrophic bacteria *Dickeya dadantii* has also been subject of study by Chl-FI. In the case of *N. benthamiana* plants, inoculations at high dose of inoculum seemed to overcome plant defense capacity, whereas plant inoculated at low dose did not show tissue maceration, and bacterial growth was inhibited. The extent and timing of changes in photosynthesis measured by Chl-FI parameters was dose-dependent, taking place earlier in the high-dose-infected leaves. Tissues surrounding inoculated areas of low-dose-infected leaves showed increased reversible NPQ as well as decreased values of F_V_/F_M_ and Φ_PSII_. Since reversible NPQ is actively controlled by the plant, it was proposed that this protective mechanism against photoinhibition was positively enhanced by the plant as part of the defense response (Pérez-Bueno et al., 2016). In the case of *D. dadantii*-melon–infected plants, Chl-FI detected decreased values of F_V_/F_M_ and NPQ in the whole leaf. The magnitude of these changes was more pronounced upon infection with high bacterial dose (**Figure 2**). In combination with multicolor fluorescence imaging and thermography, data obtained by Chl-FI were used to feed classificatory algorithms able to distinguish between healthy and infected plants at high accuracy. Those mathematical models based on data from infiltrated areas were susceptible to apply to whole leaves, offering a high performance of classification (Pineda et al., 2018). An image analysis procedure for quantifying the leaf area impacted by *Xanthomonas fuscans* subsp. *fuscans* has also been developed. F_V_/F_M_ was the parameter used to presymptomatically diagnose the infection caused by this pathogen on bean leaves. The segmentation of F_V_/F_M_ images aimed to quantify disease severity by a thresholding approach (Rousseau et al., 2013). On the other hand, *Xanthomonas oryzae* pv. *oryzae* infection (bacterial blight) in rice causes inhibition of photosynthesis and could be detected by a decrease in F_V_/F_M_, Φ_PSII_, and F_t_ (Šebela et al., 2018).

PsbS, key for the NPQ mechanism, was one of the few photosynthetic proteins that rapidly decreased in abundance in cell cultures of *Arabidopsis* after treatment with a peptide derived from the bacterial motor protein flagellin (flg22). The registered decrease in NPQ values in samples treated with flg22 was dose-dependent. Thus, NPQ was proposed to be a positive regulator of pathogen-associated molecular pattern-triggered immunity (Göhre et al., 2012). Plants overexpressing bacterial outer surface protein A in their chloroplast have also been subject of study by Chl-FI. Transplastomic lines were unable to grow autotrophically and required the supply of exogenous sugars. Therefore, they suffered from photosynthesis impairment that could be measured as decreases in both F_V_/F_M_ and Φ_PSII_ when transferred from sugar-supplemented culture medium into soil (Hennig et al., 2007).

### Fungi and Oomycetes

Among the different biotic stresses, infections caused by fungi and oomycetes are the diseases most widely studied by Chl-FI. The photosynthetic performance of both oomycetes- and fungi-infected plants usually presents complex spatial and temporal patterns (Barón et al., 2016). It is so because infected leaves usually consist of regions of cells directly colonized by the pathogen surrounded by apparently healthy areas and remote regions (Scholes and Rolfe, 1996; Osmond et al., 1998; Chou et al., 2000; Meyer et al., 2001). The way fungi and oomycetes interacts with their host plant depends on the lifestyle of the pathogen: biotrophic, necrotrophic, and hemibiotrophic.

#### Biotrophic Fungi and Oomycetes

**Figure 3:**
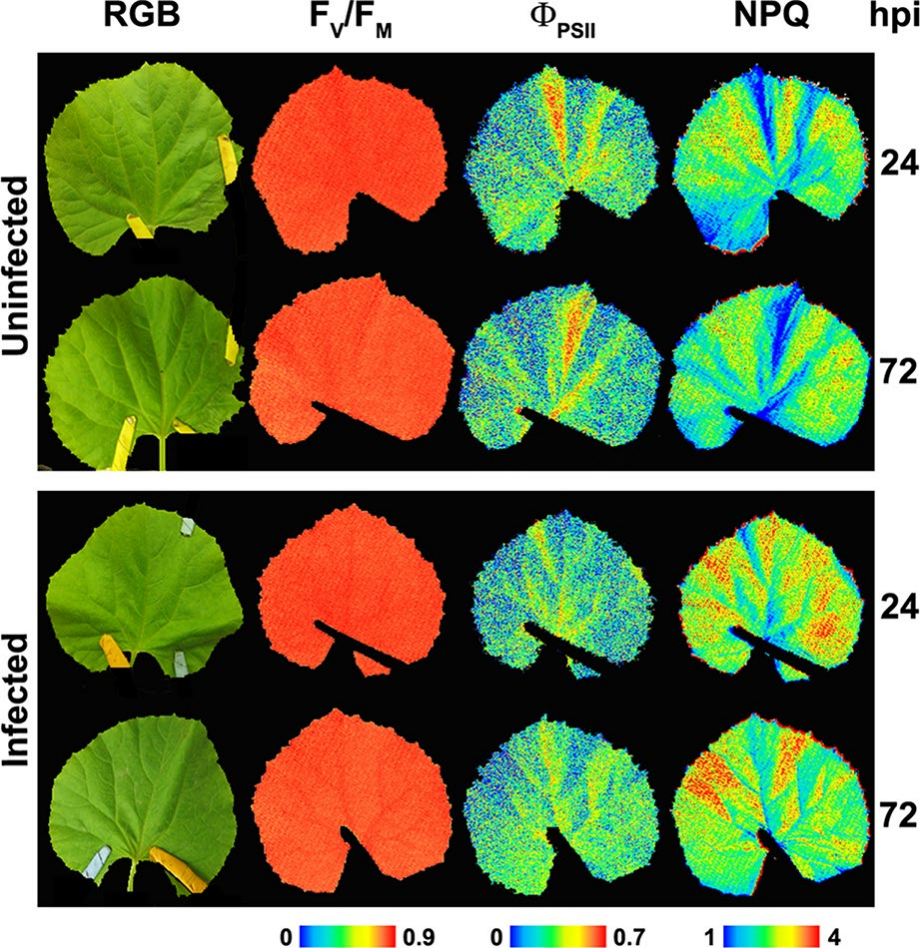
Representative images of the symptomatology (color reflectance pictures, RGB), PSII efficiency (F_V_/F_M_ and Φ_PSII_) as well as non-photochemical quenching (NPQ) from non-infected and *Podosphaera xanthii*–infected melon leaves at 24 and 72 hours post-infection (hpi). Modified from Polonio et al. (2019) (http://creativecommons.org/licenses/by/4.0/) with permissions.

Typically, biotrophic organisms lower the rate of leaf photosynthesis of their compatible hosts. Oat leaves infected with the fungus *Puccinia coronata* displayed lower Φ_PSII_ values than the controls, whereas non-qP, measured as qN, increased from 8 dpi (Scholes and Rolfe, 1996). The oomycetes *Albugo candida* progressively decreased Φ_PSII_ while NPQ increased, and F_V_/F_M_ showed no changes in *Arabidopsis* leaves (Chou et al., 2000). The oomycete *Bremia lactucae* also caused a considerable patchy decrease of F_V_/F_M_ in infected lettuce leaf discs (Bauriegel et al., 2014), as well as an increase in NPQ and a reduction in both Φ_PSII_ and F_V_/F_M_, associated to a decrease in Chl content (Prokopová et al., 2010). On the other hand, changes in Chl-FI parameters caused by the fungus *Podosphaera xanthii* in melon plants cannot be attributed to alterations in Chl content of leaves (**Figure 3**). These changes involved a decline in Φ_PSII_, while NPQ increased and F_V_/F_M_ did not display any changes in *P. xanthii*–infected melon leaves (Polonio et al., 2019). This is in accordance with previous results obtained for cucumber leaves infected with *P. xanthii*, which displayed a decrease in Φ_PSII_ several days before a reduction in Chl content could be detected (Berdugo et al., 2014). The fungus *Erysiphe cichoracearum* also caused low Φ_PSII_ and high NPQ values in oak-infected leaves relative to the controls (Repka, 2002).

F_V_/F_M_ was also used to diagnose several diseases, such as that caused by the fungus *Hemileia vastatrix* on coffee plants, since this parameter significantly correlated with visual severity of the infection (Honorato Júnior et al., 2015), and that caused by the endophytic fungus *Pestalotiopsis* spp. in cedar needles (Ning et al., 1995). Invasion of bean leaves by the rust fungus *Uromyces appendiculatus* was detected presymptomatically during the fluorescence induction kinetics as discreet areas of high Chl-F emission (coinciding with centers of subsequent lesion development) encircled by a halo of diminished emission relative to that of uninfected tissues (Peterson and Aylor, 1995). At the early stages of the *Plasmopara viticola* (oomycete)–grapevine interaction, F_V_/F_M_ and Φ_PSII_ were identified as the most sensitive presymptomatic reporters of the infection, as their heterogeneous distribution on inoculated leaves was associated with the presence of developing mycelium (Cséfalvay et al., 2009).

The interaction of another biotrophic fungus, *Blumeria graminis*, and cereals has also been studied. In the case of wheat, this fungal infection can be presymptomatically detected by Chl-FI as a reduction in F_V_/F_M_ (Kuckenberg et al., 2009). On the other hand, this fungus reduced Φ_PSII_ and increased NPQ, whereas F_V_/F_M_ only diminished in the latter stage of barley leaf infection (Brugger et al., 2018). It has been suggested that development of *B. graminis* haustoria and hyphae damages chloroplast structure in barley leaves, causing inhibition of photosynthesis (measured as Φ_PSII_) both in cells directly below fungal colonies and in adjacent cells when compared with non-inoculated leaves (Swarbrick et al., 2006).

#### Necrotrophic Fungi and Oomycetes

Regarding necrotrophic fungi, the effects of *Rhizoctonia solani* in the photosynthetic performance of rice only could be detected once the symptoms appeared. Both necrotic zone and adjoining lesion areas significantly reduced the NPQ values, whereas F_V_/F_M_ values only decayed in the lesions compared to the control tissues (Ghosh et al., 2017). Cashew seedlings inoculated with two different isolates of *Lasiodiplodia theobromae* displayed significantly lower F_V_/F_M_ values respecting to the controls, as well as decays in Φ_PSII_ and increases in NPQ values before any visual symptoms appeared. The photosynthetic perturbations were clearly noticeable in F_V_/F_M_ images along the borders of leaves, spreading gradually into inner regions (Muniz et al., 2014). In the *Bipolaris sorokiniana*–infected leaves of susceptible wheat plants, there was a progressive decrease of photosynthesis (measured as F_V_/F_M_ and Φ_PSII_) correlated to the expansion of lesions, as well as to a progressive loss of Chl (Rios et al., 2017). The impact of *Ascochyta rabiei* fungus in chickpea leaves was assessed by the impairment of Φ_PSII_ on infected leaves, since this fungus altered source/sink relationships (Esfeld et al., 1995). F_V_/F_M_ and Φ_PSII_ showed an inhibition of photosynthesis in the direct vicinity of the *Botrytis cinerea* infection sites of tomato leaves. At the same time, the primary metabolism is activated in circular areas surrounding the infection sites together with decreased values of NPQ in such areas. However, no alterations in the primary metabolism could be detected in the rest of the leaf tissue, farther away from the infection site (Berger et al., 2004). In contrast, *B. cinerea* caused a different Chl-FI spatial pattern on ice plants: while F_V_/F_M_ diminished only in the infected areas, NPQ increased in the non-infected regions of the leaves (Sekulska-Nalewajko et al., 2019). Chl-FI was applied to evaluate the extent of the impact caused by different strains of *Pythium irregulare* Buisman in ginseng plants. Values of F_V_/F_M_ significantly diminished relative to the controls only for leaves inoculated with the highest pathogenic strain assayed of this oomycete (Ivanov and Bernards, 2016). Other less common Chl-FI parameters could be good indicators of disease. That was the case of F_M_/F_0_, F’_V_/F’_M_, and F’_V_/F’_0_ for the detection at early stages of the infection of avocado trees by the soil-borne fungus *Rosellinia necatrix*. However, F_V_/F_M_ decreased dramatically only when the first symptoms appeared (Granum et al., 2015).

#### Hemibiotrophic Fungi

The interaction of several species of the hemibiotrophic fungal genus *Colletotrichum* with their host plants has also been studied by means of Chl-FI. The infection caused by *Colletotrichum orbiculare* in *N. benthamiana* plants can be visualized as presymptomatic decreases in the parameter F_V_/F_M_ but late in the biotrophic phase (Tung et al., 2013). Based on the measured reductions in F_V_/F_M_ and NPQ, and increases in the efficiency of PSII (measured as Φ_PSII_ and qP), the impact of the infection by *Colletotrichum truncatum* on the photosynthetic performance of the soybean leaflets was noticeable exclusively on the necrotic vein tissue, the only tissue colonized by the pathogen (Dias et al., 2018). Bean leaves infected with *Colletotrichum lindemuthianum* registered a decreased photosynthetic activity in terms of Φ_PSII_ in green areas only during the necrotrophic stage of the infection; such inhibition of photosynthesis was more pronounced in the dark brown necrotic lesions (Meyer et al., 2001). For the system sugar beet–*Cercospora beticola*, spots of high intensity fluorescence emission developed presymptomatically in the areas where necrotic lesions would later develop (Chaerle et al., 2004). Distribution and progression of head blight disease (caused by fungi *Fusarium* spp.) in winter wheat ears can be determined using F_V_/F_M_, since values of this parameter clearly diminished in the single grains gradually colonized within the spikelets (Bauriegel et al., 2010; Bauriegel et al., 2011).

#### Other Studies on Fungal Infections

Chl-FI has been used to distinguish between compatible and incompatible fungus/oomycete-plant interactions, or to visualize the infection effects on plants with different degrees of susceptibility to the pathogen. A pixel-wise analysis of the parameter F_V_/F_M_ could distinguish resistant and susceptible lettuce lines against the biotrophic oomycete *B. lactucae* (Bauriegel et al., 2014). Photosynthesis was reduced during an incompatible barley*–B. graminis* interaction, accompanied by an increase in NPQ. This effect was more evident in cells straight related with attempted penetration of the biotrophic fungus but also in neighboring cells (Swarbrick et al., 2006). The distribution pattern of both Φ_PSII_ and NPQ over the entire leaf of wild-type oak infected with *E. cichoracearum* is heterogeneous when compared to those displayed by a Chl-deficient mutant of oak with high resistance to this fungus. These patterns coincided with fungal distribution in the infected leaves (Repka, 2002). The photosynthetic performance of the *B. sorokiniana*–infected leaves measured in terms of F_V_/F_M_ and Φ_PSII_ was dramatically impaired on the most susceptible wheat cultivar compared to a less susceptible cultivar (Rios et al., 2017).

The effects of fungal phytotoxins directly applied on leaves or fruits of susceptible plants could also be assessed by Chl-FI. Based on Kautsky kinetic measurements, an experimental algorithm was proposed to identify affected and unaffected leaves of both *Brassica napus* and *Sinapis alba* plants treated with destruxins produced by the fungus *Alternaria brassicae* (Soukupová et al., 2003). The same method was used to detect apple areas treated with roseotoxins (phytotoxin produced by *Trichothecium roseum*) from non-treated regions (Žabka et al., 2006).

Some substances can confer certain protection against fungal infection, and Chl-FI has been proved to be useful to evaluate the photosynthetic performance of treated-infected plants. The protective effect of magnesium (Tatagiba et al., 2016a) and silicon (Tatagiba et al., 2016b) against *Monographella albescens* infection in rice plants was reported as a lower decrease of both qP and Φ_PSII_ in those infected plants treated with either Mg or Si relative to non-treated-infected plants. 2R,3R-butanediol (BD) is a volatile organic compound able to elicit induced systemic resistance (ISR), and thus, to delay 24 h the necrosis development caused by in *C. orbiculare* infection in *N. benthamiana* plants. Chl-FI was used to determine the levels of damage in BD-treated plants relative to those where ISR was not elicited. F_V_/F_M_ showed that BD treatment significantly increased the amount of healthy tissue and diminished the extent of necrotic tissue (Tung et al., 2013). F_V_/F_M_ also reported that the treatment with the fungicides epoxiconazole and pyraclostrobin decreased the symptoms produced by *H. vastatrix* on coffee leaves, since this parameter was shown to positively correlate to symptoms severity (Honorato Júnior et al., 2015).

### Pests

Pests also cause severe economic losses in crop yield around the world. It includes insects and also weeds and parasitic plants, such as the *Orobanche* genus or the *Santalaceae* family. The effect of herbivory, and parasites to a lesser extent, on host photosynthesis has been analyzed by Chl-F.

Herbivore insects devour vast amounts of plant biomass each year. However, simply considering the quantity of tissue removed may undervalue their real impact on yield production, because often insect damages affect photosynthesis in remaining leaf tissues. This “indirect” effect on primary metabolism may be considerably greater than the direct removal of leaf area (Nabity et al., 2009). The mechanisms governing the spatial patterns of photosynthesis following herbivory have been explored by Chl-FI, among other imaging techniques. In the case of *Arabidopsis* leaves affected by the first and fourth instars of the lepidopteran *Trichoplusia ni*, the measured decrease in Φ_PSII_ inversely correlated with the percentage of area removed; however, the correlation was considerably greater for the first instar. This difference in correlation slope is probably related to the different way of instars to cause photoinhibitory damage in the remaining tissues. Lower values of Φ_PSII_ and F_V_/F_M_, as well as increases in NPQ registered in leaves eaten by fourth instars, were circumscribed to a thin band immediately adjacent to the hole, whereas those values for leaves damaged by first instars were altered also in the areas between some of the holes (Tang et al., 2006). A deeper analysis of the effect of the first instar on *Arabidopsis* leaves revealed that photosynthetic damage (measured as lower levels of Φ_PSII_ and F_V_/F_M_ and increased NPQ) was most severe at the edge of holes but decreased inversely with the distance from them. Moreover, in portions of the leaf where the photosynthesis was depressed, the defense-related cinnamate-4-hydroxylase gene expression was upregulated, suggesting a trade-off between primary and secondary metabolisms (Tang et al., 2009). Moreover, the results obtained by Chl-FI highlighted the potential differences between the herbivory damages caused colony-reared and wild-caught larvae of the tobacco hornworm *Manduca sexta* in the ornamental plant *Datura wrightii*. Whereas herbivory by colony-reared larvae produces no significant changes in photosynthesis, wild larvae induced a fast and spreading decrease of Φ_PSII_ within minutes, in both eaten and uneaten leaves. NPQ was increased near the damage and increased progressively in distant areas of the leaf away from the wound (Barron-Gafford et al., 2012). Both Φ_PSII_ and F_V_/F_M_ values also resulted diminished in the case *M. sexta*–damaged *Nicotiana attenuata* leaves, but not in those leaves attacked by the *Tupiocoris notatus* mirid bugs, which displayed no alterations in the photosynthetic activity. *T. notatus* is known to render the plant more resistance to other, more damaging, herbivores (Halitschke et al., 2011). The combination of Chl-FI with other techniques also demonstrated that the inhibition of photosynthesis in *M. sexta*–damaged *N. attenuata* leaves is mediated by the jasmonic acid defense signaling pathway (Nabity et al., 2013).

Plant parasites take nutrients, including photosynthate, from their host plants. In particular, parasitic plants can be holoparasites (non-photosynthetic), such as broomrapes (*Orobanche* sp.) and witchweeds (*Striga* sp.), or hemiparasitic plants like rattle (*Rhinanthus* sp.). Parasites can affect photosynthesis in different ways, depending on the interaction established between the parasite and the host plant. Few works have analyzed such effect by single point Chl-F measurements (Strong et al., 2000; Gurney et al., 2002; Cameron et al., 2008; Rodenburg et al., 2008), and even less by Chl-FI. Rousseau et al. (2015) reported the detection of the broomrape *Orobanche ramose*–infested *Arabidopsis* plants by a decline in qP and NPQ. There is also a lack of Chl-FI works studying the effect of parasitic nematodes. It was the case of sugar beet showing photoinhibition (detected as a decrease in F_V_/F_M_) when infested by *Heterodera schachtii* (Schmitz et al., 2006).

## Biotic Stress Detection in High-Throughput Platforms and at Field Scale

**Figure 4:**
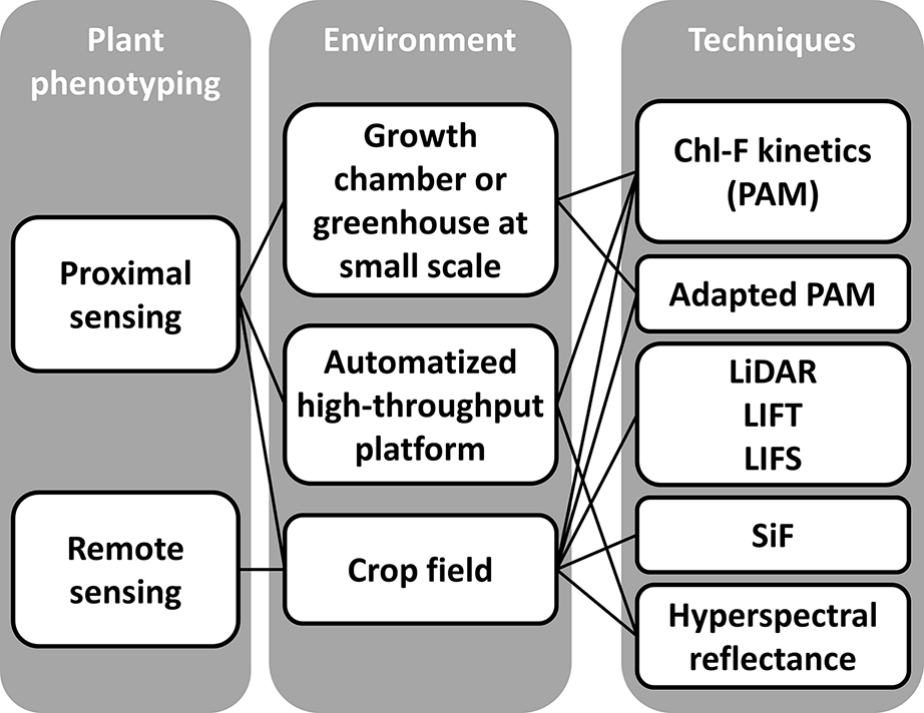
Different approaches based on chlorophyll fluorescence imaging systems to plant phenotyping. The scales of analysis include: lab scale, high-throughput platforms under controlled environmental conditions, and filed scale under natural environmental conditions. Techniques are grouped by the source of light used for the measurements: LEDs (PAM measurements), laser (LiDAR, LIFT, and LIFS), and natural light (SiF and hyperspectral reflectance on canopy surface).

Crop improvement based on plant breeding needs of accurate phenotyping (Mir et al., 2019). Thus, there is an increasing interest from research institutions to develop systems for high-throughput plant phenotyping both at greenhouse and at field scale (Roitsch et al., 2019, and references therein). Although Chl-FI provides valuable information on photosynthetic activity and general fitness of plants, this type of system is not usually used on plant phenotyping. This is partly due to some technical limitations affecting the robustness and reproducibility, as reviewed by Li et al. (2014). The Chl-F at the scale of high-throughput platforms and field can be analyzed or derived by measurements with a variety of devices that can be classified by the type of light source used—artificial or solar—and by the environment-controlled *vs*. natural conditions (**Figure 4**), as described below.

### Robotized Platforms for Plant Phenotyping Under Controlled Environmental Conditions

Automatized phenotyping platforms are developing quickly in recent years. In Europe, the European Infrastructure for Multi-Scale Plant Phenomics and Simulation (EMPHASIS), under the European Plant Phenotyping Network 2020 (ERRN2020), offers 145 facilities for phenotyping of plants under controlled conditions plus lean fields and intensive fields (https://emphasis.plant-phenotyping.eu/database). The North American High-Throughput Phenotyping (NAPPN) counts with 20 phenotyping platforms in Canada, USA, and Mexico. In the rest of the world, the main facilities are offered by the Australian Plant Phenomics Facility and the China Plant Phenotyping Network. For more complete information about worldwide available high-throughput plant phenotyping facilities/platforms and their services, please see Tschiersch et al. (2017) and Mir et al. (2019). There is also an increasing interest on low-cost (and “do it yourself”) sensor solutions (Roitsch et al., 2019). Wang et al. (2018) reported a monitoring system under controlled environmental conditions able to perform large-area Chl-FI and multispectral reflectance imaging that allows not only a continuous monitoring of crop physiology but also the possibility of implementing automatic diagnosis of drought, nutrition deficiency, and infection with *B. cinerea*. In spite of the interest in phenotyping plants by Chl-FI in automatized platforms under controlled environmental conditions, there is a lack of studies addressing the effects of biotic stress on host-plant photosynthesis by such approach.

### Remote and Proximal Sensing for Plant Stress Detection Under Natural Environments

Active Chl-F measurements have conventionally been measured upon excitation with artificial lighting systems, generally by lamps or LEDs. However, technical limitations make very difficult the measurement of Chl-F remotely, mainly for the analysis of quenching kinetics. This problem has been partially solved by the application of systems such as: laser-imaging detection and ranging systems (LiDAR), laser-induced fluorescence transients (LIFTs), and laser-induced fluorescence spectroscopy (LIFS). Alternatively, passive methodologies based on sun-induced fluorescence (SiF) have been developed to analyze photosynthetic activity at leaf and canopy level. Finally, the analysis of hyperspectral reflectance indices correlating with Chl-F parameters seems a promising tool. These sensors can be implemented on a wide range of remote sensing systems: (i) stationary, with sensors mounted on cranes, towers, or cables; (ii) vehicle-based sensors; (iii) robotic devices; (iv) drones (unmanned aerial vehicles, i.e., UAVs) or airplanes; and (v) moreover, satellite imagery could also be used to cover very large areas. However, the use of satellite sensors has been limited to regional scales due to their low spatial resolution, making this approach not feasible for most crops (Gago et al., 2015; Smigaj et al., 2019).

#### Imaging of Chlorophyll Fluorescence Excited by Artificial Light Systems

Several studies have analyzed the photochemical activity under biotic stress in experimental plots by imaging PAM fluorometers. The infection with *Phyllosticta* fungus on *Quercus velutina* and *Cercis canadensis* trees inhibited Φ_PSII_ on areas surrounding infection points without evidence of compensation for this decrease in the remaining tissue (Aldea et al., 2006b). On the contrary, the inhibition of PSII in areas of redbud and sweetgum trees affected by *Cercospora* was counteracted by an increase in the photosynthetic efficiency in the undamaged leaf tissue and in a halo surrounding lesions (McElrone et al., 2010). The effect of herbivory on photosynthesis has been analyzed in several studies. Aldea et al. (2006b) investigated the indirect effect of herbivory on Chl-F by performing feeding trials with the Polyphemus caterpillar *Antheraea polyphemus* Cramer. This work concluded that, of all classes of damage studied, galls had the largest halos of depressed PSII activity when normalized to the size of visible injury. Nabity et al. (2012) compared the indirect effect of different types of herbivory on photosynthesis of aspen and birch trees. This work showed that the PSII activity was inhibited in the undamaged tissue and concluded that elevated ambient CO_2_ was related to a decrease in the transpiration rate of leaves and could indirectly reduce the effects of herbivory on photosynthesis. On the other hand, P. viticola caused a decrease in FV/FM in infected spots of susceptible grapevine leaves. Symptomatic and asymptomatic regions over a leaf could be discriminated by the spatial distribution of FV/FM (Šebela et al., 2014). Furthermore, the most destructive fungus for rice crops worldwide, Magnaporthe oryzae, could be detected as a decrease in Φ_PSII_ and F_t_ (Šebela et al., 2018). Other Chl-FI devices adapted for remote sensing can be found in the literature. Such is the case of the CropReporter, a CCD camera (coupled with LEDs to induce Chl-F transients) onboard the platform Field Scanalyzer (Virlet et al., 2017). This sensor allows monitoring plant growth, morphology, physiology, and plant fitness under natural conditions.

Alternatively to lamps and LEDs, other systems excite Chl-F by laser. Saito et al. (2002) reported a method for imaging Chl content on trees based on fluorescence measured by a LiDAR system. This approach can be used to analyze spatial distribution of green tissues providing a detailed 3-D model of the canopy. Furthermore, the photosynthetic activity can be measured at the whole plant and canopy level by scanning methods that use LIFTs, as previously reviewed by Omasa et al. (2006) and Fiorani et al. (2012). A terrestrial-adapted LIFT (mounted on a telescope or on a tower above the canopies) has demonstrated its potential in remote measurements of photosynthetic traits in cottonwood and oak trees (Kolber et al., 2005); avocado trees (Rascher and Pieruschka, 2008); limes, oaks, and pines (Pieruschka et al., 2014); as well as in barley and sugar beet (Raesch et al., 2014). The results were comparable to those obtained at leaf scale by the PAM system. Moreover, Osmond et al. (2017) developed a prototype of LIFT that induces a Chl-F transient by a series of short flashes in a saturation sequence and operates from up to 2-m distance under ambient light, allowing the analysis of photosynthetic regulation at canopy scale during sun flecks in natural environments. More recently, a methodology using a portable LIFS system in combination with classifying algorithms allowed the presymptomatic detection of citrus trees affected by citrus greening, also called huanglongbing (Ranulfi et al., 2016). Although the use of laser-based measurements is well-established on remote sensing, the automated data processing and analysis need further development—for example, taking in account the effects of complex heterogeneous canopy structures on Chl-F parameters (Pieruschka et al., 2014; Roitsch et al., 2019).

#### Sun-Induced Fluorescence

A different approach for remote assessment of photosynthesis activity is based on remote measurements of steady-state Chl-F (noted either as Ft or Fs) induced by the sun, since it yields strong correlations with stomatal conductance and net CO_2_ assimilation rate (Flexas et al., 2002). The SiF is retrieved from narrow spectral bands, whereas conventional PAM fluorescence is measured over broad spectral bands. Moreover, unlike PAM fluorescence, SiF is affected by environmental light but can be applied at leaf, canopy, and regional scale. The setups and methodologies for measuring SiF have been intensively reviewed by Aasen et al. (2019). The development of methods to measure steady-state SiF has been mainly focused on improving the retrieval of valid fluorescence values under natural illumination that correlate firstly with data obtained by conventional fluorometers and secondly with parameters related to the CO_2_ fixation rate.

SiF, emitted by Chl *a* in the broad-band-red and far-red regions of the spectrum near 683 and 736 nm, respectively (Joiner et al., 2016; Goulas et al., 2017), can be analyzed by hyperspectral cameras. Garzonio et al. (2017) developed an UAV system equipped with small hyperspectral cameras to measure the visible and near-infrared (VNIR) surface reflectance and SiF. This setup retrieved fluorescence in absolute units with a good spatial resolution. However, most works apply methods that can retrieve Chl-F at leaf to canopy level from data collected by high-resolution spectrometers. The Fraunhofer line depth (FLD) method, described by Plascyk and Gabriel (1975), has been used successfully to estimate SiF, demonstrating its correlation with Chl content or Φ_PSII_ (Tubuxin et al., 2015). Moreover, at seasonal time scales, SiF correlated with the electron transport rates (ETR) and constitutive heat dissipation (YNO) in avocado and orange jasmine orchards (Wyber et al., 2017). Several modifications to the method have been developed by other authors along the years. Thus, Maier et al. (2003) and Ni et al. (2015) published a modification of FLD based on three spectral bands named FLD3. Rascher et al. (2015) and Pinto et al. (2016) presented a new methodology based on FLD3 to estimate Φ_PSII_, which was verified against the standard PAM method. SiF quantified by FLD3 was significantly associated with gross primary production (GPP) at ecosystem scale (Zarco-Tejada et al., 2013) and with leaf-level measurements of CO_2_ assimilation (Zarco-Tejada et al., 2016). Indeed, Guanter et al. (2014) concluded that SiF data could contribute to improve global models for more accurate projections of agricultural productivity and also to estimate climate impact on crop yields.

A step forward in the use of SiF for the estimation of GPP could be taken based on Goulas et al. (2017), who reported that SiF in the far red (FRSiF) yields a stronger correlation than the traditionally used SiF in the red region (RSiF) of the spectrum. Moreover, FRSiF provided a better estimation of GPP than greenness-based indices (Damm et al., 2015). These findings were corroborated by other authors (Joiner et al., 2016; Campbell et al., 2019). Similarly, Yang et al. (2018) used a singular vector decomposition method for the spectral range of 745 to 780 nm as a proxy for absorbed PAR (APAR) in rice paddies at diurnal and seasonal time scales. Also, Miao et al. (2018) found a strong and positive correlation between SiF and APAR for soybean. Nonetheless, it was suggested that only SiF correlates with ETR and the photosynthetic yield at large spatial scales (Du et al., 2017). The estimation of GPP based on SiF would need to take into account relevant environmental information to model the light use efficiency of photosynthesis (Yang et al., 2018), canopy structure, and competing energy pathways (Damm et al., 2015).

These SiF retrieval methods have been applied not only to UAV data but also to satellite data, including GOSAT, GOME-2, OCO-2, SCIAMACHY, and TanSat, as reviewed recently by Ni et al. (2019). Furthermore, the fluorescence explorer project (FLEX), currently held by the European Space Agency, is aiming to map vegetation fluorescence at high spatial resolution, leading to better insight into plant health and stress.

A different approach for stress detection could take advantage of the combination of SiF with different techniques. Rahimzadeh-Bajgiran et al. (2017) reported a FLD and laser-induced saturation pulse (FLD-LISP) method as a robust and accurate technique for the estimation of Chl-F parameters such as Φ_PSII_, NPQ, and ETR, for several plant species. In parallel, a model based on measurements of two satellite detectors (SiF from Orbiting Carbon Observatory-2 and surface reflectance from the moderate resolution imaging spectroradiometer [MODIS]), provided a very sensitive indicator of drought (Zhang et al., 2018).

Until now, few studies have addressed the study of infections in the field by SiF. Hernández-Clemente et al. (2017) proposed a 3-D modelling approach that improved the correlation between the F_S_ at leaf level from ground data measurements and the image-based fluorescence inferred by FLD3. This work studied an oak forest (*Quercus ilex*) affected by water stress and *Phytophthora* infection. Their results lead to the conclusion that their model could make possible to map SiF for single-tree assessment of forest physiological condition offering the possibility of early disease detection. Raji et al. (2015) analyzed the infection of cassava plants by the *Cassava mosaic virus* by the FLD method, finding that the fluorescence ratio F687/F760 showed a good correlation with net photosynthesis rate, the Chl content, and the laser-induced Chl-F (LICF) ratio F685/F735 and that this parameter could be a good marker of early stress detection in crops and vegetation.

#### Reflectance Spectra for the Estimation of Chlorophyll Fluorescence Parameters

Measurements of Chl-F under natural environments are technically challenging, mainly by the difficulty of splitting fluorescence from reflectance. Strong efforts have been made to develop an alternative to Chl-F detection valid for remote sensing, based on the analysis of hyperspectral leaf reflectance spectra and derived vegetation indices (VIs). Hyperspectral reflectance is an imaging technique that allows the detection of plant stress in a non-invasive and objective way (Thomas et al., 2018).

Several VIs have been found to provide a good estimate for the efficiency of PSII on a wide range of plant species. The single ratio (SR) and the curvature index (CUR) were highly correlated with F_V_/F_M_ (Jia et al., 2016). Peng et al. (2017) concluded that normalized difference vegetation index (NDVI) correlated with F_V_/F_M_ only in non-stressed plants. In contrast, other authors reported no significant correlation between NDVI and Chl-F parameters (Jia et al., 2016). More promising is the estimation of Φ_PSII_ by VIs. PRI correlates positively with this Chl-F parameter at canopy level (Gamon et al., 1992; Naumann et al., 2008) and at the leaf level for different plant species (Gamon et al., 1997). The ETR could be also estimated by PRI at leaf scale; however, a PRI calibrated for the pigments content provided an even better estimation (Rahimzadeh-Bajgiran et al., 2012). Other VIs, such as the normalized multi-band drought index (NMDI) or the water band index (WBI), have also been found to correlate with F_V_/F_M_ and Φ_PSII_ for a range of alpine sward species (Kycko et al., 2019). Furthermore, the maximum daily photosynthetic rate correlated with NDVI and SR (Gamon et al., 1995).

The capacity for energy dissipation at the PSII could also be estimated by several VIs across plant species. NPQ and the de-epoxidation state of the xanthophyll cycle (which controls NPQ) correlate negatively with PRI at canopy (Gamon et al., 1992) and leaf scale (Gamon et al., 1997; Van Gaalen et al., 2007; Rahimzadeh-Bajgiran et al., 2012; Alonso et al., 2017; Sancho-Knapik et al., 2018). More recently, the indices ΔPRI (which accounts for pigment composition in PRI) and the normalized difference spectral index (NDSI) were reported to estimate NPQ more accurately than PRI (Maimaitiyiming et al., 2017; Kováč et al., 2018).

Particularly in recent years, several research groups have established correlations between new VIs and Chl-F parameters. The so-called fluorescence ratio indices R_690_/R_600_ and R_740_/R_800_ (being Rx the leaf reflectance at the wavelength x) were able to qualitatively track the leaf F_S_ in a grapevine canopy (Dobrowski et al., 2005). Zarco-Tejada et al. (2009) could retrieve the Chl-F signal from reflectance airborne measurements on olive, peach, and orange orchards, demonstrating the correlation between the retrieved Chl-F and in-field F_S_ measurements, the derivative index D_702_/D_680_ (being the Dx the derivative of the reflectance at the wavelength x), and the reflectance indices R_690_/R_630_, R_761_ – R_757_, and R_761_/R_757_. Similarly for rice plots, the parameters F_0_, F_M_, and F_V_/F_M_ could be monitored by the indices (R_680_−R_935_)/(R_680_ + R_935_) and R_680_/R_935_, while Φ_PSII_ and NPQ could be estimated by the ratios (R_800_ − R_445_)/(R_800_ − R_680_) and (R_780_ − R_710_)/(R_780_ − R_680_), respectively (Zhang et al., 2011). In *Suaeda salsa* experimental plots, indices (R_680_ − R_935_)/(R_680_ + R_935_) and R_680_/R_935_ correlated with F_V_/F_M_, Φ_PSII_, and qP, whereas (R_780_ − R_710_)/(R_780_ − R_680_) correlated with NPQ (Zhang et al., 2012). Moreover, the first derivative of some spectral indices such as D_705_/D_722_ and D_730_/D_706_ strongly correlated with F_V_/F_M_ (Jia et al., 2016). Also, indices using long-wave red edge and near-infrared reflectance (NDRE740 and CI740) probed to be adequate for the estimation of F_V_/F_M_ (Peng et al., 2017).

The correlation between the VIs and the Chl-F parameters is however influenced by different factors (canopy structure, environmental conditions such as light, wind, etc.), limiting the scaling up from leaf to canopy level (Thomas et al., 2018). The resolution of the cameras and, in particular, the methodology for the analysis of data need to be improved in order to obtain more robust and accurate estimations of photosynthetic activity, GPP, and detection of plant stress for precision agriculture (Camino et al., 2018; Cendrero-Mateo et al., 2018).

## Conclusions and Future Outlook

Precision agriculture and plant breeding need of high-throughput imaging techniques to reach the main goal of a sustainable low-input management of crops. On this matter, Chl-FI is a very relevant technique, as a sensitive tool for monitoring crop performance and detection of plant stress. This approach has been applied extensively at lab scale on its own and also complemented with other imaging techniques and omics approaches. The implementation of Chl-FI to high-throughput scale, and particularly under natural light conditions, presented technical challenges partially solved by adapted imaging systems based on lamps, LEDs, or lasers. For high-throughput phenotyping, alternative techniques based on solar light might be advantageous over fluorescence imagers, obtaining high temporal and spatial resolution data. It includes the development of methodologies to retrieve SiF or to estimate photosynthetic parameters by hyperspectral reflectance as a more feasible alternative to Chl-FI in high-throughput platforms and crop fields for plant biotic stress detection and monitoring. However, up to date, there is still a lack of standardized methods to obtain Chl-F data showing high correlation with photosynthetic and crop yield traits under natural conditions. Ongoing research into this area will contribute to set up widely adopted standards that could be applied across crop species and agrosystems.

Nevertheless, the application of SiF and hyperspectral reflectance on plant phenotyping need of further research on the effects of environmental factors (i.e., incoming PAR, viewing solar geometry, direct-to-diffuse light ratios, air temperature, and wind), canopy heterogeneity, and architecture on the remote measurements. In this sense, advances have been made in the last year, developing 3D models of canopies at different spatial resolutions by a range of methodologies (Gastellu-Etchegorry et al., 2017; Janoutova et al., 2019; Roitsch et al., 2019). Particularly in the case of hyperspectral reflectance, the applicability of spectral indices for predicting photosynthetic activity and GPP should be explored across different canopy types and temporal and spatial scales.

In the future, the routine programs to monitor crop fields should include Chl-F sensors but, most importantly, should make use of SiF and hyperspectral imagery, along with the widely used thermography to reach the final goal of a low-input highly efficient agriculture with minimal impact in the environment. Furthermore, high-throughput plant phenotyping platforms need of further development to overcome the economic constraints in their use, and to make of them easy-to-use reliable tools. Thus, low-cost and do-it-yourself platforms could be the best solution to achieve a real impact on agricultural yields and natural environment protection across the world.

## Author Contributions

MP-B, MP and MB contributed to the writing up.

## Funding

This work was supported by grants from CICE-Junta de Andalucía (P12-AGR-0370) and Ministerio de Ciencia, Innovación y Universidades (RTI2018-094652-B-I00).

## Conflict of Interest Statement

The authors declare that the research was conducted in the absence of any commercial or financial relationships that could be construed as a potential conflict of interest.

## Abbreviations

APAR, absorbed photosynthetically active radiance; BD, 2R,3R-butanediol; cfu, colony forming units; Chl, chlorophyll; Chl-F, chlorophyll fluorescence; Chl-FI, chlorophyll fluorescence imaging; CMV, *Cucumber mosaic virus*; dpi, days post-inoculation; ETR, electron transport rate; Φ_PSII_, effective quantum yield of photosystem II; FLD, Fraunhofer line depth; FLD3, Fraunhofer line depth based on three spectral bands; F_0_, minimum fluorescence in the dark-adapted state; F’_0_, minimum fluorescence in the light-adapted state; F_M_, maximum fluorescence in the dark-adapted state; F’_M_, maximum fluorescence in the light-adapted state; FRSiF, far-red solar-induced fluorescence; F_S_, chlorophyll fluorescence at the light-adapted steady state; F_t_, current fluorescence in the light-adapted state; F_V_/F_M_, maximum quantum yield of photosystem; GPP, gross primary production; hpi, hours post-inoculation; HR, hypersensitive response; ISR, induced systemic response; LICF, laser-induced chlorophyll fluorescence; LiDAR, Laser imaging detection and ranging; LIF, laser-induced fluorescence; LIFS; laser-induced fluorescence spectroscopy; LIFT, laser-induced fluorescence transients; NDVI, normalized difference vegetation index; NPQ, non-photochemical quenching; PAM, pulse-amplitude modulation; PAR, photosynthetically active radiance; PRI, physiological reflectance index; PSII, photosystem II; qN, non-photochemical quenching; qP, photochemical quenching; ROS, reactive oxygen species; RSiF, red solar-induced fluorescence; SiF, solar-induced fluorescence; SR, single ratio; TCV, *Turnip crinkle virus*; TMV, *Tobacco mosaic virus*; UAV, unmanned aerial vehicle; UCP, uncoupling protein; VI, vegetation index.
